# Integrated DNA methylome and transcriptome analysis reveals the ethylene-induced flowering pathway genes in pineapple

**DOI:** 10.1038/s41598-017-17460-5

**Published:** 2017-12-07

**Authors:** Jiabin Wang, Zhiying Li, Ming Lei, Yunliu Fu, Jiaju Zhao, Mengfei Ao, Li Xu

**Affiliations:** 1 Institute of Tropical Crop Genetic Resources, Chinese Academy of Tropical Agricultural Sciences, Danzhou, 571737 Hainan China; 2 Ministry of Agriculture Key Laboratory of Crop Gene Resources and Germplasm Enhancement in Southern China, Danzhou, 571737 Hainan China; 3 Hainan Province Key Laboratory of Tropical Crops Germplasm Resources Genetic Improvement and Innovation, Danzhou, 571737 Hainan China

## Abstract

Ethylene has long been used to promote flowering in pineapple production. Ethylene-induced flowering is dose dependent, with a critical threshold level of ethylene response factors needed to trigger flowering. The mechanism of ethylene-induced flowering is still unclear. Here, we integrated isoform sequencing (iso-seq), Illumina short-reads sequencing and whole-genome bisulfite sequencing (WGBS) to explore the early changes of transcriptomic and DNA methylation in pineapple following high-concentration ethylene (HE) and low-concentration ethylene (LE) treatment. Iso-seq produced 122,338 transcripts, including 26,893 alternative splicing isoforms, 8,090 novel transcripts and 12,536 candidate long non-coding RNAs. The WGBS results suggested a decrease in CG methylation and increase in CHH methylation following HE treatment. The LE and HE treatments induced drastic changes in transcriptome and DNA methylome, with LE inducing the initial response to flower induction and HE inducing the subsequent response. The dose-dependent induction of *FLOWERING LOCUS T-like* genes (*FTLs*) may have contributed to dose-dependent flowering induction in pineapple by ethylene. Alterations in DNA methylation, lncRNAs and multiple genes may be involved in the regulation of *FTLs*. Our data provided a landscape of the transcriptome and DNA methylome and revealed a candidate network that regulates flowering time in pineapple, which may promote further studies.

## Introduction

Pineapple (*Ananas comosus L*.) is an economically significant tropical fruit of the bromeliads. Natural flowering in pineapple is commonly induced by short-day photoperiods, cool night temperatures and dry weather, and other stresses, such as root damage, can also trigger flowering^1^. Ethylene-induced flowering is widely used to synchronize pineapple flowering, facilitating year-round production.

The transition from vegetative growth to flowering is a crucial developmental change for flowering plants. This transition occurs in response to various environmental and endogenous cues. In Arabidopsis, flowering time is controlled by age-, autonomous-, sugar budget-, gibberellin-, ambient temperature-, photoperiod- and vernalization-dependent pathways^2–4^. Floral integrator genes such as *FT*, *SOC1*, and *AGL24*, converge various cues and activate the floral identity genes (e.g., *LFY*, *AP1*, *SEP3* and *FUL*), which confer the transition to the floral meristem^5^.

Ethylene is a plant hormone that regulates plant growth and development, as well as responses to biotic and abiotic stresses^6,7^. The role of ethylene in the flowering transition is unclear, as ethylene can both promote and delay flowering in both Arabidopsis and rice under different conditions^8–11^. In pineapple, flowering is triggered by a burst of ethylene production in response to various cues, in which the *AcACS2* plays an important role^12^. Four ethylene receptors genes (*AcERS1b*, *AcERS1b*, *AcETR2a*, and *AcETR2b*) have been cloned, and expression analysis showed that *AcERS1b*, *AcETR2a*, and *AcETR2b* play key roles in pineapple flowering^13^. Ectopic overexpression of *AcPISTILLATA (PI)* and *AcFT* induces early flowering in Arabidopsis; however, *PISTILLATA* and *FT* expression peaked on day 40 after ethylene treatment when the fruit and floral organs were forming, indicating an important role in floral organ and fruit development^14,15^. Liu *et al*. (2016) utilized Illumina sequencing to integrate transcriptomic changes shoot apical meristems of floral buds in response to ethylene^16^, indicating that *LTI, FT*, and *VRN1* involved in the process of floral development. The ethylene level of plants were increased and the GA_3_ level were decreased during first 24 hours after flowering forcing using ethephon, coupling with the upregulation of *GA2ox1* and *PI* ^17^. And 48 hours after treatment, the shoot apex showed differentiation signs and plants can been seen “open heart” 2 weeks after treatment^1,17^. However, further research is needed to understand the molecular mechanism regulating the transition from vegetative to reproductive growth of pineapple during forcing flowering.

DNA methylation is a widely studied epigenetic modification that occurs at CG, CHG (H = A, T or C) and CHH sites through the covalent addition of a methyl group to cytosine in plants. DNA methylation plays a key role in plant development and stress responses^18–22^. Moreover, DNA methylation is involved in controlling flowering time. Silencing of the *FWA* gene by DNA methylation controls flowering time in *Arabidopsis* ^22^. Treatment with 5-azaC, a cytosine methyltransferase inhibitor, induces flowering in Arabidopsis, wheat and *Pharbitis nil* ^23–25^.

With the publication of the genomes of the pineapple varieties F135 and MD-2 genome, mapping genomic DNA methylation and transcriptomic changes in inducing flowering is now possible^26,27^. After treating plants with water, a low concentration of ethephon (600 mg/L) and high concentration of ethephon (1200 mg/L) 24 hours, we utilized PacBio RS II and Sequel iso-seq, Illumina short-reads sequencing and whole-genome bisulfite sequencing (WGBS) to track changes in the transcriptome and DNA methylome, which triggering the floral transition. The results implied that the DNA methylation level, alternative splicing (AS), alternative polyadenylation (APA) and the expression levels of multiple genes were changed in response to ethylene, which may contribute to ethylene-induced flowering.

## Results

### Transcriptome sequencing using iso-seq and isoform detection

To detect transcriptomic changes during flowering induction, four libraries with <1-, 1-2, 2-3, and >3 k fragment sizes were constructed and sequenced using PacBio RS II and Sequel, yielding 1,068,545 ROI reads in total (Supplementary Table S1). Of the ROI reads, 774,999 were 5′ primer observed reads, 1,081,410 were 3′ primer observed reads, 856,009 were poly A tail reads and 725,933 were full-length non-chimeric reads. Using Quiver, the full-length reads were polished and classified as 122,338 high-quality and 234,917 low-quality reads (Supplementary Table S2).

**Figure 1:**
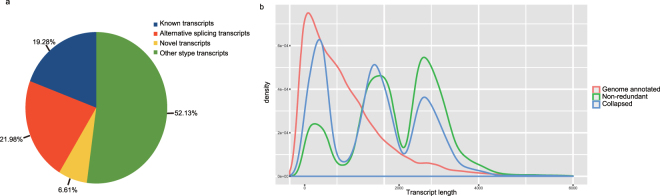
Summary of high-quality transcripts obtained using iso-seq. (**a**) Distribution of high-quality transcripts. (**b**) Length distribution of annotated pineapple genes and high-quality transcripts. Gene models: Pineapple genome annotated gene; Non-redundant transcripts: high-quality transcripts perfectly mapped to the genome; un-collapsed transcripts: transcripts unmapped or insufficiently mapped to the genome.

After polishing, 122,338 high-quality reads were mapped to the genome using GMAP, with 99.16% of reads mapped. Then, the reads mapped to the genome with high quality were collapsed into non-redundant transcripts, which resulted in 45,876 transcripts. Figure 1b shows the length distribution of annotated genes in the pineapple genome, perfectly mapped transcripts, and insufficiently mapped and unmapped transcripts, with mean lengths of 1,171, 1,857 and 1,695 bp, respectively. Using Cuffcompare, the mapped reads were compared to the annotated gene model. The reads mapped to 22,676 loci, including 7,673 (33.90%) novel loci. Through AS analysis, we identified 29,801 isoforms from 13,264 loci, of which 9,371 isoforms matched the intron chain of genome annotation genes and 20,140 alternative splicing isoforms (Fig. 1a). A total of 71 transcripts mapped to 34 miRNA genes, and 174 transcripts mapped to 39 phasiRNA genes. An additional 352 transcripts mapped to 140 transposable elements (TEs), and 726 reads mapped to rRNA genes.

### Transcriptome sequencing using Illumina short reads

Sequencing identified 26,870,796, 29,999,998, 27,224,962, 29,526,890, 29,688,904, 26,878,052 30,487,590, 30,085,504, and 29,442,846 bp for the 9 samples of the CK, LE and HE groups, with three biological replicates each (Supplementary Table S3). The clean reads had a 59.05% genome-map rate and a 47.29% gene-map rate. Using the short reads, we detected the expression of 23,030 (89.36%) annotated genes.

### Alternative splicing and alternative polyadenylation

**Figure 2:**
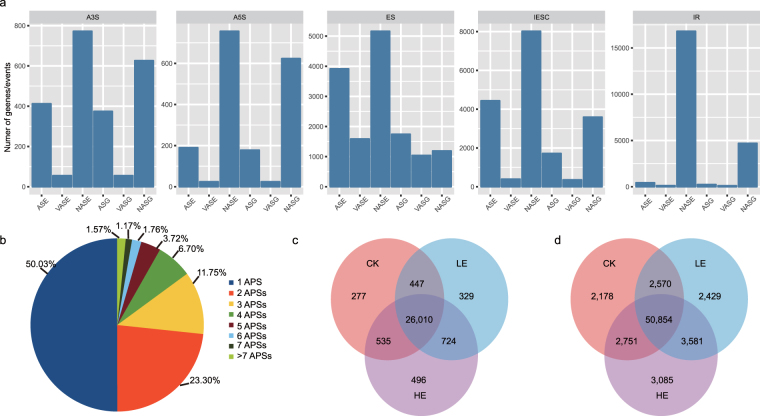
Summary of alternative splicing isoforms and alternative adenylation isoforms. (**a**) Statistics of the numbers of alternative splicing isoforms of genes. A3S: alternative 3′ splice site; A5S: alternative 5′ splice site; ES: exon skipping; IESC: internal exon size change; IR: intron retention. ASE: alternative splicing events annotated in pineapple genome; VASE: the iso-seq reads validated alternative splicing events annotated in pineapple genome; NASE: the new alternative splicing events arose; ASG: ASE related genes; VASG: VASE related genes; NASG related genes. (**b**) Statistics of the number of alternative adenylation isoforms of genes. (**c**) The distribution of alternative splicing events. (**d**) The distribution of alternative splicing isoforms in CK, LE and HE. (**e**) The distribution of alternative adenylation isoforms in CK, LE and HE.

Of the 28,901 isoforms, AS events were divided into five categories: 1) 827 transcripts with a 3′ AS site; 2) 780 transcripts with a 5′ AS site; 3) 6,746 transcripts with exon skipping; 4) 16,917 transcripts with intron retention; and 5) 8,408 transcripts with sizes of exon disagreement (Fig. 2a). A total of 7,767 genes had more than one AS isoform (Fig. 2b). Given the APA of mRNA, 6,604 of 13,216 loci had more than one alternative polyadenylation site (APS). Using short-read quantification, we identified 277 CK-specific, 329 LE-specific and 496 HE-specific AS isoforms, with 2,178 CK-specific, 2,429 LE-specific and 3,085 HE-specific isoforms (Fig. 2c and d).

The pineapple genome contains 1,601 annotated transcription factor (TF) loci^28^. The iso-seq reads mapped to 780 loci with 1,629 isoforms. Of the TF loci, 352 loci have multiple isoforms. A total of 12 CK-specific, 21 HE-specific and 22 LE-specific isoforms, as well as 96 ethylene-induced isoforms, were detected. A total of 2,568 APS of 619 loci were identified, with 74 CK-specific, 103 LE-specific and 125 HE-specific isoforms, as well as 329 ethylene-induced APS isoforms.

### Novel transcripts and lncRNAs

**Figure 3:**
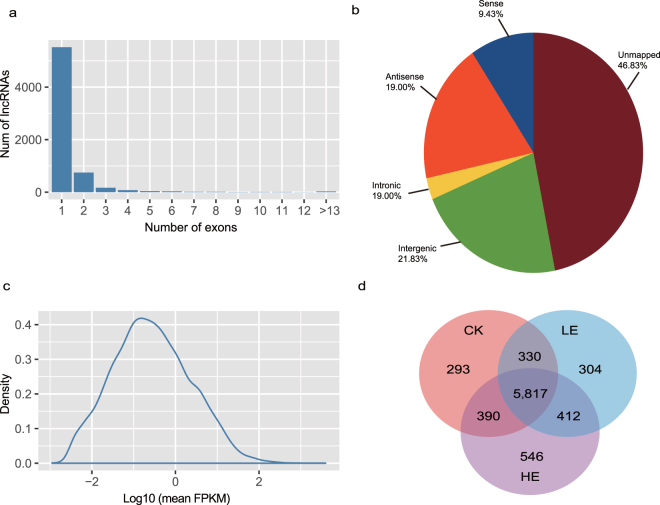
Summary of lncRNAs. (**a**) The distribution of the number of exons in lncRNAs. (**b**) The distribution of five types of lncRNAs. (**c**) The mean expression level lncRNAs. The expression level were normalized use Log10 (mean FPKM). (**d**) The distribution of lncRNAs in CK, LE and HE.

By predicting the coding potential of novel transcripts, we obtained 12,536 candidate lncRNAs and 8,090 candidate novel coding transcripts. The novel coding transcripts were aligned to the NR database, with 846 transcripts hitting known genes (Supplementary Dataset S1). According their position relative to the gene annotations, the lncRNAs were classified into five groups: 1,182 (76.00%) in the sense strand, 2,382 (6.28%) in the antisense strand, 365 (2.70%) in intronic regions, 2,736 (7.25%) in intergenic regions and 5,871 (7.76%) unmapped or insufficiently mapped to the genome (Fig. 3b). The lncRNAs ranged in length from 280 to 6,025, with a mean length of 1,860. Of the lncRNAs mapped pineapple genome, 5,517 lncRNAs had a single exon, 745 lncRNAs had two exons and 405 lncRNAs had more than two exons (Fig. 3a). LncRNAs were expressed in a lower level with median 0.69 and mean 21.74 FPKM value (Fig. 3c). And 3,700 lncRNAs were with 0 FPKM values, which were not detected by short reads. Using the quantification of short reads, we identified 293, 304, and 504 CK-, LE- and HE-specific lncRNAs, respectively (Fig. 3d). To determine the function of lncRNAs involved in flowering induction, we predicted the candidate targets of 298 lncRNAs in *cis*. The flowering related genes *FTL1*, *BHLH93*, *GA2OX8*, *MYB30* and *EIL1* were targets of lncRNAs.

### Differential expression analysis

After quantification of short reads using RSEM, we identified differentially expressed genes (DEGs) and transcripts using NOISeq. We identified 935, 833 and 799 upregulated and 674, 710 and 846 downregulated genes in CK_vs_LE, CK_vs_HE and LE_vs_HE, totaling 2,978 genes. We also performed differential expression analysis on protein-coding mRNA isoforms and lncRNAs. In total, 4,647 isoforms from 2,655 loci were differentially expressed in response to ethylene, with 993, 1,430, 1166 upregulated and 915, 1490, 1199 downregulated isoforms in CK_vs_LE, CK_vs_HE and LE_vs_HE, respectively. Among lncRNAs, 61, 87 and 68 were upregulated, and 56, 91 and 72 were downregulated, totaling 294 lncRNAs. From the DEGs, we selected 18 protein-coding mRNAs and 14 lncRNAs for qRT-PCR validation. The expression patterns of most genes were similar to the expression patterns quantified using short reads (Supplementary Figs S1 and 2).

### Co-expression network analysis

**Figure 4:**
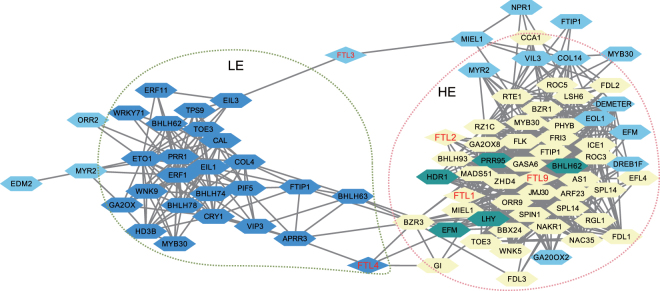
The co-expression network of flowering-related genes. FTLs are shown in red. The different colors of the nodes represent gene clusters: yellow, clusters showing a positive response to HE; green, clusters showing a negative response to HE; steelblue, clusters with a positive response to LE; blue, clusters with no significant response to HE or LE.

Using WGCNA, we predicted the co-expression network of the annotated pineapple genes, and the genes were classified into 96 clusters. The interactions of each cluster were predicted using SPINE (Supplementary Fig. S3). LE and HE separately interacted with 11 clusters each. Six clusters (‘darkolivergreen4’, ‘darkturquoise’, ‘navajowhite1’, ‘paleturquoise’, ‘plum2’ and ‘saddlebrown’), containing 677 genes, negatively interacted with HE, and 5 clusters (‘pink’, ‘skyblue’, ‘skyblue3’, ‘turquoise’, and ‘yellow4’), containing 5852 genes, positively interacted with HE, representing the genes responding HE. Seven clusters (‘blue’, ‘brown’, ‘darkturquoise’, ‘darkmagenta’, ‘firebrick4’, ‘green’ and ‘skyblue’), containing 5716 genes, positively interacted with LE, and four clusters (‘coral1’, ‘purple’, ‘steelblue’ and ‘violet’), containing 802 genes, negatively interacted with LE, representing the genes responding to LE. The ‘darkturquoise’ cluster interacted positively with LE but negatively with HE. And the clusters ‘blue’, ‘pink’, ‘grey60’, ‘magenta’ interacted negatively with CK, representing the genes whose expression changed under LE or HE treatment. A subnetwork of candidate flowering genes comprising 87 nodes and 728 edges was extracted (Fig. 4 and Supplementary Dataset S2). Genes in the ‘brown’ and ‘blue’ nodes positively interacted with LE, genes in the ‘turquoise’, ‘pink’, ‘yellow4’ and ‘skyblue3’ nodes positively interacted with ‘HE’, and genes in the ‘paleturquoise’ and ‘saddlebrown’ nodes negatively interact with ‘HE’, while the else genes negatively interact CK. The networks included 5 *FTLs*, which may be regulated by the other genes in the network. *FTL4* positively interacted with LE, while *FTL1*, *FTL2* and *FTL9* positively interacted with HE. Only *FTL1* was expressed in CK, while *FTL1*, *FTL2* and *FTL9* were induced by HE. Homeostasis among these factors represses and promotes flowering, which ensures the repression of *FTL1*, *FTL2* and *FTL9* and the induction of *FTL4* in LE and CK and the induction of *FTL1*, *FTL2* and *FTL9* and the repression of *FTL4* in HE. The candidate genes involved in the regulation of TFLs may relate to the circadian rhythm and stress responses. The genes in the subnetwork can be divided into three categories: genes whose expression was more significantly altered with LE than with HE; genes whose expression was more significantly altered with HE than with LE; genes whose expression was altered by HE and LE but did not differ significantly between the two. In other words, changes in the expression of genes in the three categories in LE and HE may be successive processes that ultimately results the induction of *FTL4*. According to the DISCERN score, the level of topological alternation of gene networks in different conditions, *TOE3* (LOC109711971), *NAC35* (LOC109713884) and *JMJ30* (LOC109725417) were the top-scoring genes in LE_vs_HE that were involved in *FTL* repression.

### Landscape of genomic DNA methylation

Three pineapple samples under CK, HE, LE treatment was subjected to WGBS. The results generated 29,592,403,200, 27,528,550,600, and 22,905,508,800 bp of clean data, covering 85.36%, 85.07%, and 85.75% of the genome for CK, HE, and LE. Furthermore, 84.78% (83.67% CG, 85.21% CHG, 84.65% CHH), 83.44% (82.59% CG, 84.28% CHG, 83.48% CHH), and 85.26% (84.07% CG, 85.62% CHG, and 85.48% CHH) of Cs were mapped with reads in the CK, LE and HE groups.

**Figure 5:**
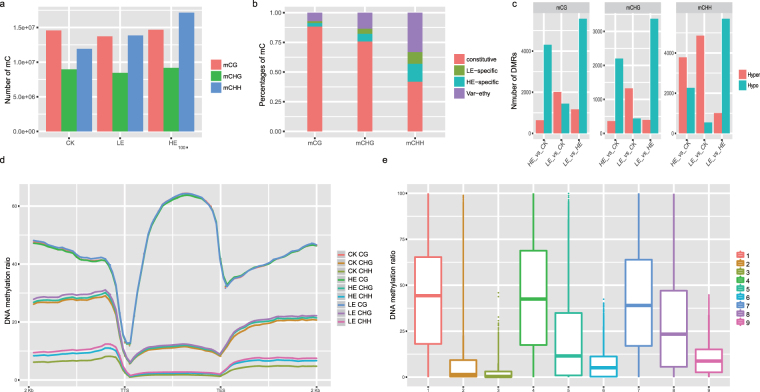
Summary of DNA methylation. (**a**) Statistics on the numbers of mCs. (**b**) The distribution of mCs in CK, LE and HE. (**c**) The statistics of DMRs. (**d**) The mean DNA methylation level of in the different gene regions. (**e**) The box-plot of DNA methylation level in the different gene region. 1: CG methylation in gene upstream; 2: CHG methylation in gene upstream; 3: CHH methylation in gene upstream; 4: CG methylation in gene downstream; 5: CHG methylation in gene downstream; 6: CHH methylation in gene downstream; 7: CG methylation in gene body; 8: CHG methylation in gene body; 9: CHH methylation in gene body.

In total, 14,584,760, 13,721,730 and 14,685,198 mCG; 8,957,987, 8,455,848 and 9,182,888 mCHG; and 11,916,174, 13,859,070 and 17,146,463 mCHH were detected in the CK, LE and HE groups, respectively (Fig. 5a). These results suggested that LE and HE differentially altered genomic DNA methylation patterns. The numbers of mCGs and mCHGs decreased following LE treatment but increased following HE treatment. The increase in the number of mCHHs was also much higher in HE than in LE.

The mCs in each context were further classed as constitutive, LE-specific, HE-specific and varied (Fig. 5b). Constitutive methylated mC decreased in the order of mCG, mCHG and mCHH, indicating that the pathways mediating mCG, mCHG and mCHH methylation responded differentially to HE and LE.

Furthermore, to detect the effects of DNA methylation changes on genes, the average methylation level of the upstream, gene body and downstream were calculated (Fig. 5e). The DNA methylation level decreased in order of CG, CHG and CHH. In upstream, the CHG and CHH methylation level is lower than in gene downstream and body. Compared with CK, mCHG and mCHH increased in the HE and LE groups, and mCG increased with LE and decreased with HE, indicating that ethylene treatment significantly altered the average methylation level (Fig. 5d). In particular, mC levels in the 5′ and 3′ UTR was more significantly altered than other gene regions.

According the mean methylation level of gene body and upstream, the genes were classed into 4 clusters: Cluster 1 contained genes that had high level CG methylation in upstream; cluster 2 contained genes that had higher CG, CHG and CHH methylation level in gene body than in upstream; cluster 3 contained genes that had lower methylation level in general; cluster 4 contained genes that had high level CG and CHG methylation in both gene body and upstream, but low CHH methylation in gene body and upstream (Supplementary Fig. S4A–D). The 4 clusters had 7209, 7277, 6878 and 2275 genes, separately, of which the mean expression level of cluster 4 was significantly lower than other clusters (Supplementary Fig. S4E).

### DMR identification and stats

Using methylPipe, DMRs between CK and LE (CK_vs_LE), between CK and HE (CK_vs_HE), and between HE and LE (LE_vs_HE) were identified. A total of 4,941, 2,563 and 6047 DMRs were identified for CK_vs_HE, 3,458, 1,764 and 5,462 for CK_vs_LE, and 6,740, 3,769 and 6,714 for LE_vs_HE in the contexts of CG, CHG and CHH (Fig. 5c). It’s thought that DMRs for CK_vs_LE represented the initial alterations in flowering inducing, LE_vs_HE represented for the subsequent alterations and CK_vs_HE represented the ultimate alterations that required for flowering. In total, 4,210, 2,156 and 5,316 DMRs in contexts of CG, CHG and CHH arose in CK_vs_HE but not in CK_vs_LE, and 734, 305 and 1,071 DMRs in contexts of CG, CHG and CHH arose in both CK_vs_HE and CK_vs_LE, which were the flowering inducing related DMRs. About 50% DMRs in the contexts of CG and CHG are overlapped, indicating the co-regulation of DNA methylation in the contexts of CG and CHG.

Along with DMRs for CG and CHG in CK_vs_HE, the number of hypo DMRs exceeded the number of hyper DMRs, suggesting that ethylene treatment prefers hypo methylation during flowering induction, while the DMRs for CHH prefers hyper methylation. In addition, approximately 33.31%, 3.38% and 63.30% of DMRs were distributed in genes, TEs and intergenic regions. In total, the upstream region of 4,336 genes and the coding region of 3,684 genes showed overlapping DMRs in the mCG, mCHG and mCHH contexts in response to HE and LE.

### The regulation of FT-like genes

**Figure 6:**
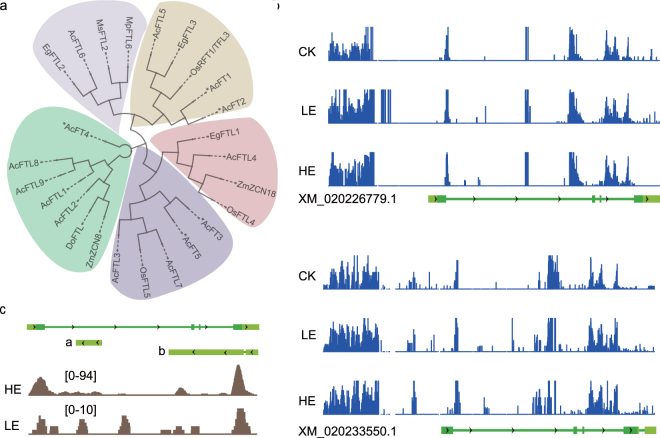
Summary of FTLs. (**a**) Phylogram of different FLT-like genes from monocots. Ac: Ananas comosus; *Ac: Allium cepa; Os: Oryza sativa Japonica Group; Mp: Musa acuninata AAA Group; Ms: Musa AAB group; Eg: Elaeis guineensis; Zm, Zea mays; Do: Dichanthelium oligosanthes. (**b**) The CHH methylation type of FTL1 transcripts XM_020233550.1 and FTL9 transcripts XM_020226779.1. (**c**) The location and expression of two lncRNAs from FTL9. a: the lncRNA c10180/f1p1/463; b: the lncRNA c19722/f1p1/1757.

Nine *FT-like* genes were annotated in the pineapple genome. Phylogenetic analysis of these *FT* genes and FT-like genes from other organisms divided the 9 *FT-like* genes into five groups (Fig. 6a). *FTL1*, *FTL2*, *FTL8* and *FTL9* were grouped with *ZmZCN8*, which functions as a flowering activator^29^. *FTL5* was positioned in the group containing *OsRFT1*, *AcFT1 and *AcFT2, which also function as flowering activators^30,31^. *FTL4* is positioned in the group containing *ZmZCN18*, which is constitutively expressed in leaves^29^. *FTL8*, which does not have a complete ORF, is predicted to be a lncRNA^32^.

We observed the CHH methylation level were increased in *FTL1* and *FTL9* gene upstream and body upon LE treatment (Fig. 6b). But the CHH methylation of *FTL9* was decreased upon HE treatment compared to LE treatment. For the last exon region of *FTL1*, CG methylation level was increased upon HE treatment. The significantly different DNA methylation alteration may contribute to the different expression pattern of *FTL1* and *FTL9*.

lncRNA prediction identified two antisense lncRNAs (c10180/f1p1/463 and c19722/f1p1/1757) from the *FTL9* locus (Fig. 6c) and one antisense lncRNA (c494294/f1p1/2025) from the *FTL4* locus. The lncRNA c10180/f1p1/463 was induced by both HE and LE, while the expression of c19722/f1p1/1757 was higher in HE than in LE. The expression of c494294/f1p1/2025 increased in LE and decreased in HE. The three antisense lncRNAs may be involved in regulating *FTL9* and *FTL4* in *cis*.

## Discussion

The iso-seq results provided a comprehensive landscape of full-length isoforms. Using the obtained 122,338 HQ reads, we identified 28,901 isoforms of the annotated gene model. Given isoforms with different APS, we identified 6,604 loci with one or more APS. Using short-reads quantification, we identified CK, LE and HE-specific AS and APA isoforms, which implied changes in the complexity of the transcriptome in response to LE and HE treatments. Through differential expression analysis of AS and APA isoforms, we identified some isoforms of genes that had different expression patterns from those of other isoforms, which may have different functions. Importantly, the identified novel 8,090 genes and the isoforms of 7,756 loci had no transcripts that equally matched to the annotated transcripts, which indicated great improvement of the gene model annotations.

lncRNAs are major components of the transcriptome and play a key role in different biological processes. In particular, lncRNAs have also been shown to participate in flowering by mediating the epigenetic silencing of *FLC* in vernalization^33^. In our study, we predicted 12,536 candidate lncRNAs, which is close to the number of candidate lncRNAs in maize (12,226)^34^. Of the 2,357 candidate lncRNAs annotated in pineapple, 458 lncRNAs we identitied in our studies. For low-expression lncRNAs that were filtered out for differential expression analysis, we identified 294 differentially expressed lncRNAs. Using WGCNA we predicted *cis* targets of lncRNAs, and the results suggested that lncRNAs may be involved in regulating genes that play a key role in ethylene-induced flowering. Further studies is needed to uncover the functions of the lncRNAs whose expression changed by ethylene treatment.

DNA methylation has been extensively studied in eukaryotes. DNA methylation is highly dynamic in response to environmental and endogenous cues. In rice, the DNA methylation level was increased by drought stress, and 70% of the changes were reset by removal of the drought stress^35^. Nitrogen deficiency can induce heritable alterations in DNA methylation to enhance tolerance among progeny in rice^36^. Ethylene is a plant hormone that regulates biotic and abiotic stress tolerance in plants^6^. Therefore, ethylene treatment may alter DNA methylation, as confirmed by our results. Compared with CK, the mean mCG and mCHG methylation level decreased in HE and but increased in LE, and mCHH methylation increased in both HE and LE. DMR analysis revealed that the upstream regions of 4,336 genes and the coding regions of 3,684 genes had overlapping DMRs in the mCG, mCHG and mCHH contexts in response to HE and LE. Moreover, in LE_vs_HE, the majority of DMRs were downregulated. For the genes in gene co-expression network, we identified 1,827, 1,705 and 412 genes interacted with HE, LE and CK separately overlapped with DMRs. In particular, for the candidate regulators in flowering related subnetworks, we identified 28 genes were overlapped with DMRs, including *FTL1*, *FTL9*, *FTIP1*, *NAKR1*, *etc*., of which the expression alternation may resulted by DNA methylation alteration during flowering inducing (Supplementary Dataset S3). Our results indicated that DNA methylation was differentially altered in response to HE and LE, which may play key role in ethylene-induced flowering.

Of the nine *FT-like* genes that were annotated in the pineapple genome, *FTL1*, *FTL2*, and *FTL9* were induced by HE and LE. *FTL4* was increased in LE and decreased in CK_vs_HE. Moreover, *FTL6* was not expressed in our study but is known to be expressed in fresh and flower organs, indicating a role for *FTL6* in floral organ development^14,16^. Being increased in CK_vs_HE, *FTL1*, *FTL2* and *FTL9* may play overlapping or distinct roles in promoting flowering. Conversely, *FTL4* was decreased in CK_vs_HE and may function as an antiflorigen. Our findings therefore support a balance between florigen and antiflorigen activity during flowering, as elucidated in tomato^37^.

After DEG analysis, we identified multiple genes related to flowering that may act as regulators of *FTLs*. The DEGs included homologs of genes that promote flowering, such as *SPL14*, *BHLH93* ^38^, *MYB30* ^39–41^, *Flowering BHLH* (*BHLH78, BHLH62 and BHLH74*)^42^, and *AS1* ^43^, as well as homologs of genes that repress flowering, such as *SPIN1* ^44^, *RAV1* and *RAV* ^45^, *JMJ30 and EFM* ^46^, *NAC35* ^47^ and *APETALA2* ^48^. In pineapple, *SPL14-like* is a homolog of *OsSPL14* and *AtSPL9*, with *OsSPL14* regulating tiller and panicle branching and *AtSPL9* regulating flowering by increasing miR172^49^. *NaKR1* and *FTL9* display similar expression patterns, regulating the long-distance translocation of *FT* ^50^. *FTIP1*, which contributes to the movement of FT from companion cells to sieve cells^51^, was also upregulated in CK_vs_HE. The upregulation of *BHLH93*, *SPL14*, *AS1* and *MYB30* and downregulation of *JMJ30* and *EFM* in LE_vs_HE suggested these genes may play positive roles in pineapple flowering. Otherwise, *TPS9* was upregulated in CK_vs_HE, along with the differential expression of *SUS1*, *Carbonic anhydrase*, *phosphoenolpyruvate carboxylase*, *phosphoenolpyruvate carboxylase kinase*, indicating the sugar pathway genes are also involved in flower induction.

In pineapple, flowering is triggered by a burst of ethylene production in response to various cues^12^. Ethylene treatment also triggers the biosynthesis of endogenous ethylene. The ethylene biosynthesis gene *ACS7*and *ACO1* were upregulated after treatment, and the upregulation of *ACO1* was also described by *Espinosa et al*.^17^. In the meanwhile, ethylene receptor genes *ETR2* and *ETR3*, ethylene signaling genes *EIL1* and *EIL2* and ethylene responsive transcription factors (*ERF1*, *ERF2*, *ERF3 etc*.) were upregulated. The promoter regions of *FTL1* and *FTL9* contain ethylene response element (ERE)-boxes, and ethylene signaling may directly induce *FTL1* and *FTL9* expression. As the upregulation of homologs of auxin biosynthesis gene *WEI2* and *WEI8* ^52,53^, gibberellin (GA) biosynthesis genes *GA2ox8*, abscisic acid (ABA) biosynthesis genes *VP14* ^54,55^, salicylic acid (SA) signaling regulator *NPR1* ^56^ and the jasmonic acid (JA) signaling repressor *JAZ1* ^57^ in both CK_vs_LE and CK_vs_HE, ethylene treatment may also alters JA, ABA, GA and auxin signaling or biosynthesis, which may be involved in flowering induction. The *GA2ox8* were also upregulated in LE_vs_HE, indicating that the GA_3_ level reduced with the ethylene level increased during early stage of flowering inducing^17^. Besides, the *GA2ox1* and *ACS1* were not differently expressed as described by previous study, for the reason of difference of pineapple cultivars.

AS and APA increase the complexity of the transcriptome. HE and LE treatment induced specific isoforms and, meanwhile, the expression of some isoforms of genes was also altered. The transcripts of *JAZ1* were terminated within the last exon, resulting in truncated transcripts lacking the Jas domain, which may have a contrary function in full-length transcripts in flowering timing^58^. Three alternative splice isoforms of *FTIP1* were identified with two, three and four exons, and the isoforms with four exons was specifically induced by HE. Moreover, only the isoforms with specific APS of *ACS1* was specifically induced by ethylene treatment.

In Arabidopsis, *REPRESSOR OF SILENCING1* (*ROS1*) and *DEMETER* function as bifunctional DNA glycosylases/lyases and can activate DNA demethylation^59,60^. *ROS4/IDM1* is involved in regulating active DNA demethylation^61^. The upregulation of homologs of *ROS1*, *DEMETHER*, and *IDM1* by ethylene treatment suggests that ethylene may affect DNA methylation by regulating these enzymes. In addition to *FTLs*, many putative flowering-related genes, such as the homologs of *CAL, NAC35, BHLH62, BHLH63, BHLH74, MIE1, PRR1, PRR95, APPR3, ARF23, SPL14, MYB30, PHYB, G2OX8*, had overlapping DMRs, which may be involved in the regulation of these genes. In the meanwhile, the homolog of *FTIP1* and *NAKR1* that was responsible for the transport of *FT*. So, the DNA methylation alteration may participate in the regulation of flowering by regulated the activation and transport of *FTLs*.

lncRNAs may also directly regulate *FTL* expression. As shown in Fig. 6c, the antisense lncRNAs from *FTL4* and *FTL9* may also regulated the other *FTLs* in pineapple genome, for the highly similarity of the sequences of *FTLs*. Furthermore, the DNA methyltransferase *DMT1*, *MYB30* that may directly activate *FT* expression, gibberellin biosynthesis gene *GA2OX8* and ethylene signaling gene *EIL1* were predicted to be targeted by lncRNAs in *cis*. For the limit information of pineapple genome, the functions of the most lncRNAs were unclear, some of which may directly or indirectly regulate *FTLs*.

In our study, LE treatment did not induce pineapple flowering, whereas HE treatment can induce flowering sufficiently. Similarly, in *Guzmania lingulata*, another bromeliad, plants treated with ethylene treatment for 4 h (no flowering) and 6 h (flowering) showed sharply different results^62^. Thus, flowering induction is thought to require a signaling threshold. LE treatment did not induce the threshold level of ethylene response factors. Continuous LE treatment can also induce flowering, and LE treatment is thought to induce the initial response, while HE induces the subsequent response. Through differential expression analysis and co-expression analysis, we detected the key flowering-related genes and their co-expression network, in which *FTLs* may play a central role, while the *LFY* and *SOC1* were not differently expressed. The dose-dependent induction of *FTLs* may be a key message that contributes to dose-dependent flowering induction. Through differential methylation region analysis, we also detected thousands of DMRs that contribute to dose-dependent flowering induction. As flowering integrators, *FTLs* may be controlled by multiple flowering promoters and repressors, including protein-coding genes, lncRNAs and DNA methylation.

## Conclusion

In the present study, we applied iso-seq and Illumina short-reads sequencing for transcriptome analysis. Analysis of AS and APA using iso-seq reads unveiled the complexity of the transcriptome and improved the existing gene annotation of pineapple. WGBS revealed alterations to the DNA methylation landscape of pineapple in response to HE and LE. Two of the nine annotated *FTL* genes were differentially expressed in pineapple and may play distinct roles in ethylene-induced flowering. Altered DNA methylation, lncRNAs and multiple flowering-pathway genes may be involved in the regulation of *FTLs* in the early stage of flowering inducing. Our data will facilitate further studies of the mechanism of ethylene-induced flowering.

## Methods

### Plant materials and treatment

The pineapple cultivar ‘Tainon 16’ were planted with an accession number 1–77 in an experimental area of the Institute of Tropical Crop Genetic Resources, Chinese Academy of Tropical Agricultural Sciences (CATAS). Plants were treated with 20 mL of 600 mg/L ethephon solution, 20 mL of 1200 mg/L ethephon solution, or water as a control (CK). Treatments were carried out in triplicate. The white tissue of ‘D’ leaves and stem apex were sampled 24 h after treatment and immediately frozen in liquid nitrogen. The frozen tissues were sent to BGI Life Tech Co., Ltd (Shenzhen, China) for sequencing.

### Bisulfite-treated library construction and sequencing

Genomic DNA was extracted from each sample using the CATB method^63^. DNA was fragmented to a mean size of 250 bp by sonication using a Bioruptor (Diagenode, Belgium). Using an Illumina TruSeq DNA Sample Prep Kit, the DNA fragments were then processed by repairing 3′ ends, adenylating 3′ ends, and ligating adaptors according to the manufacturer’s instructions. The ligated DNA was converted with bisulfite using the EZ DNA Methylation Gold kit (ZYMO). Then, the different-sized inserts were excised from 2% TAE agarose gels. The products were purified using the QlAquik Gel Extraction kit (Qiagen), followed by PCR amplification. Finally, sequencing was performed on an Illumina Hiseq^TM^ 4000 with paired-end 150-bp reads.

### Bisulfite sequencing data analysis

Raw bisulfite sequencing data were filtered by removing adaptor sequences, low-quality reads and contamination. The clean data were aligned to the pineapple genome using BSMAP^64^. After removing duplicate reads, the mapped reads were merged to calculate the counts of each potentially methylated cytosine. Differentially methylated regions (DMRs) in CG, CHG, and CHH contexts were detected using methylPipe, with default parameters for plants^65^.

### RNA sample preparation, library construction and sequencing

Total RNA was extracted using a Trizol kit (Promega, USA) according the manufacturer’s instructions. Total RNA was then treated with RNase-free DNase I (Takara Bio, Japan) to remove DNA. After quality verification using a 2100 Bioanalyzer and RNase-free agarose gel electrophoresis, the libraries were processed according the manufacturer’s instructions of the Truseq. 2 RNA sample prep kit (Illumina, Inc. San Diego, CA, USA). The mRNA was isolated using oligo-dT beads (Qiagen) and the Poly (A) Purist^TM^ Kit (Ambion, now Life Technologies). After quality verification on the 2100 Bioanalyzer, the products were fragmented and reverse transcribed to cDNA fragments using a PrimeScript 1st Strand cDNA Synthesis Kit (Takara). The products were then processed by end repair and adaptor ligation and subjected to sequencing using Illumina Hiseq^TM^ 4000.

The first- and second-strand cDNA were synthesized using the SMARTer PCR cDNA Synthesis Kit (Clontech) and Phusion High-Fidelity DNA Polymerase (NEB). Then, the size of the cDNA was selected with BluePipple (Sage Science), followed by normalization using the Trimmer-2 cDNA Normalization Kit (Evrogen) and amplification. Four fractions with normalized cDNA with sizes of <1, 1-2, 2-3, and >3 kb were processed to SMRT cell libraries using the DNA Template Prep Kit (Pacific Biosciences of California, Inc.). Following binding of V2 primers and SA-DNA polymerase to the templates, the complexes were then bound to magnetic beads for sequencing on PacBio RS II and Sequel. Libraries with cDNA sizes <1 and >3 kb were sequenced with two cells and the other libraries with one cell on Sequel. For low-yield libraries with <1 kb-size cDNA, the libraries were also sequenced on PacBio RS II with three cells.

Using the SMART Analysis Server 2.2.0 (Pacific Biosciences of California, Inc.), the raw data were filtered to generate reads of insert (ROIs), followed by classification of ROIs as full-length and non-full-length reads. Full-length reads were clustered using Iterative Clustering for Error (ICE) Correction and subsequently polished using Quiver. The polished full-length and high-quality sequences were mapped to the genome using GAMP^66^. Using Cuffcompare^67^, the reads were compared to the genome annotation, and then AS isoforms and APA isoforms were obtained. Then, the coding potentiality of the remaining reads was predicted using CPC^68^, CPAT^69^ and PLEK^54^. According the results, novel coding and non-coding transcripts were predicted. In addition, reads that mapped to miRNA and phasi-siRNA loci were also extracted.

To predict lncRNA targets, we predicted interaction among lncRNAs and genes and the interaction with a score >0.2 were retained. LncRNA located within the 10 kb upstream and 100 kb downstream of gene and interacted with gene in the co-expression network were supposed to target at the gene.

### Network analysis

The gene co-expression network was inferred using WGCNA^55^. Based on the gene expression data, gene clusters and relationships among clusters were detected use SPINE^70^. The network was visualized with Cytoscape v3.4. Genes were prioritized based on the DISCERN score to determine which genes contributed to topological changes of the gene-regulator-dependence network^71^.

### qRT-PCR validation

We selected 18 protein-coding genes and 14 lncRNAs for qRT-PCR validation. The primers were designed with PRIMER 6.0 software (University of Plymouth) and listed in Supplementary Table S4 and Table S5. The Actin-2 (XM_020227265.1) was used as an endogenous control. For each sample, the reaction mixture for qRT-PCR consisted of 5 μL of 2 × SYBR Green Master Mix Reagent (Applied Biosystems), 0.2 μM gene-specific primers and 50 ng of the cDNA sample. The amplification reactions were performed as follows: 95 °C for 10 min and 45 cycles of 95 °C for 5 s and 60 °C for 30 s. The relative expression of each gene was calculated using the 2−ΔΔCt method. qRT-PCR was performed with 3 biological replicates and 3 technical replicates for each experiment.

### Availability of data and materials

Raw sequences of this study were deposited in NCBI SRA database SRP111498. The iso-seq produced high-quality sequences are available in accession SRR5816603. The Illumina short-reads are available in accession SRR5816604- SRR5816611 and SRR5816613 and WGBS produced reads are available in accession SRR5816601- SRR5816602 and SRR5816613.

## Electronic supplementary material

Supplementary information

Dataset 1

Dataset2

Dataset3
