# Supplementary material for: Integrated DNA methylome and transcriptome analysis reveals the ethylene-induced flowering pathway genes in pineapple
Source: Sci Rep. 2017 Dec 7;7:17167. doi: 10.1038/s41598-017-17460-5 (PMC5719354; doi:10.1038/s41598-017-17460-5)
Supplement: Supplementary file 1 — Supplementary information [file 41598_2017_17460_MOESM1_ESM.pdf]

Supplementary Information

Integrated DNA methylome and transcriptome analysis reveals the ethylene-induced flowering pathway genes in pineapple

Jiabin Wang[123]†, Zhiying Li [123]†, Ming Lei[123],Yunliu Fu[123], Jiaju Zhao[123], Mengfei Ao[123], Li Xu [123*]

1 Institute of Tropical Crop Genetic Resources, Chinese Academy of Tropical Agricultural Sciences, Danzhou 571737, Hainan, China

2 Ministry of Agriculture Key Laboratory of Crop Gene Resources and Germplasm Enhancement in Southern China, Danzhou, 571737, Hainan, China

3 Hainan Province Key Laboratory of Tropical Crops Germplasm Resources Genetic Improvement and Innovation, Danzhou 571737, Hainan, China

*Corresponding author

Email: xllizhiying@vip.163.com

†These authors contributed equally to this work.

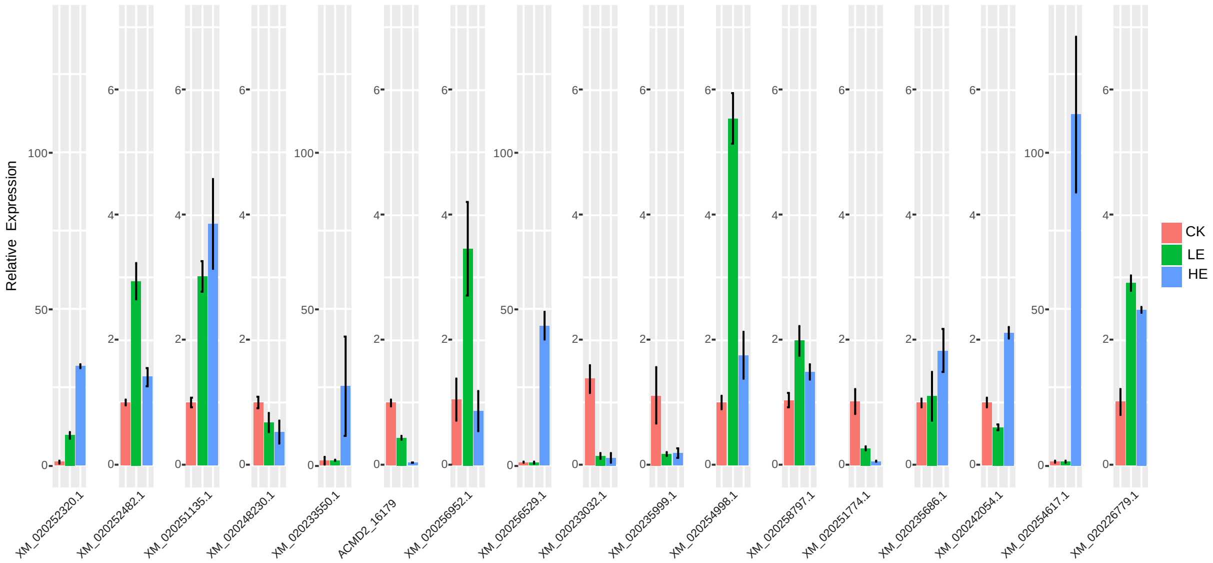

Supplementary Figure S1 qRT-PCR validation of differentially expressed genes.

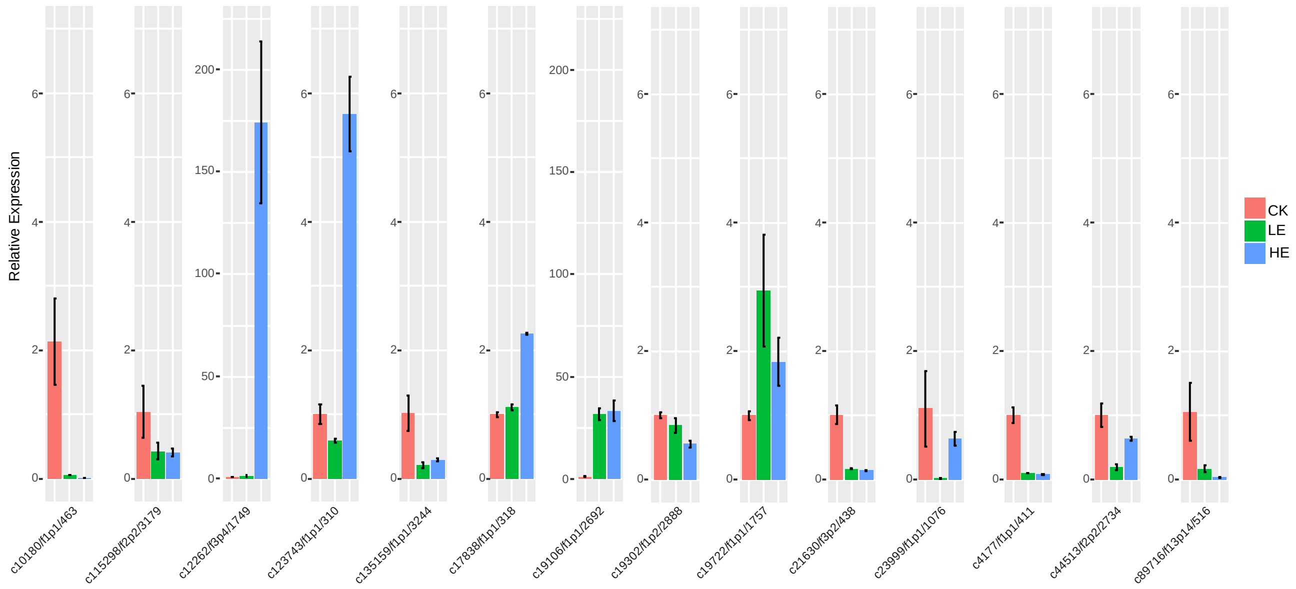

Supplementary Figure S2 qRT-PCR validation of differentially expressed lncRNAs.

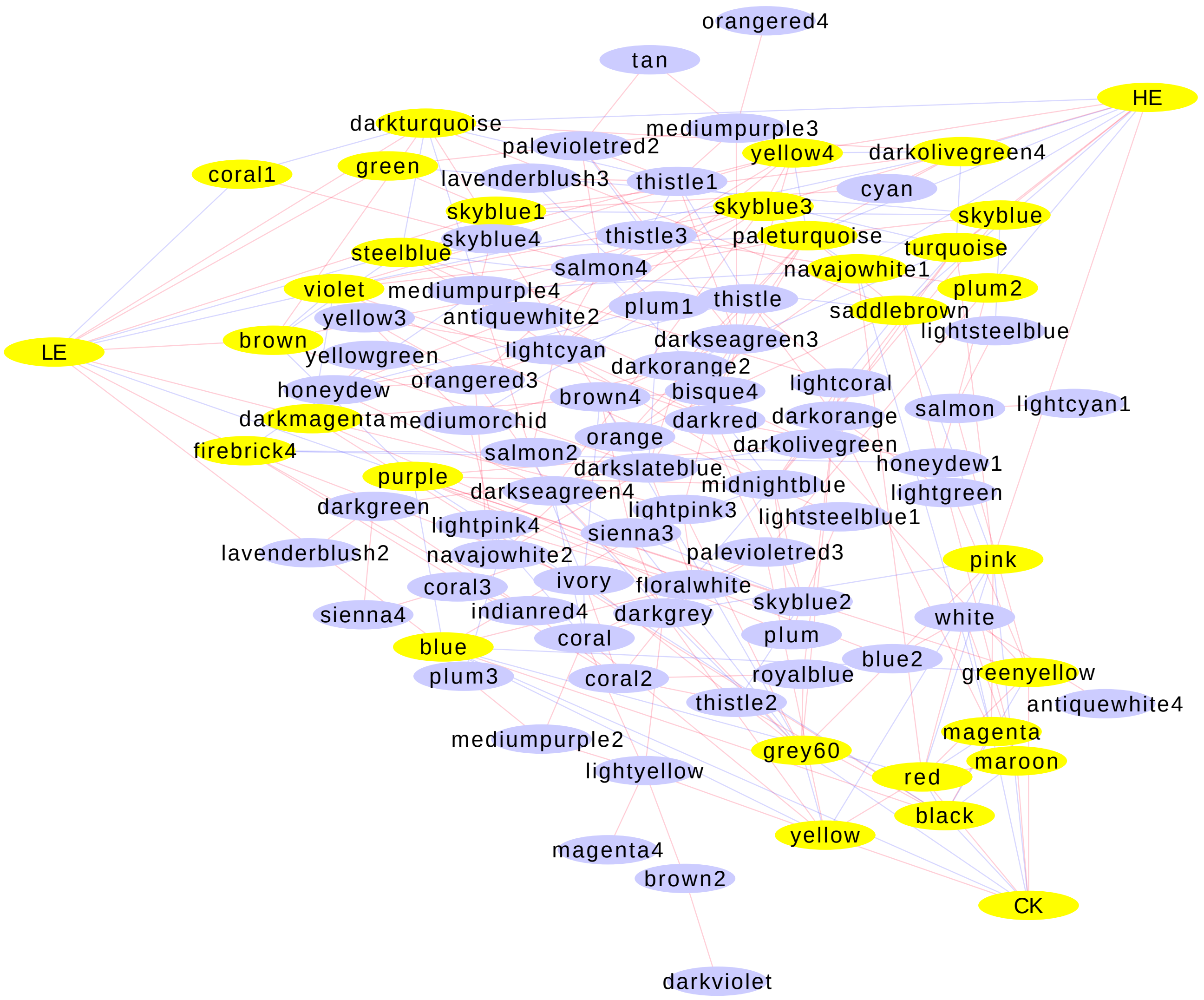

Supplementary Figure S3 The interaction network of clusters. Edges in red represent positive interactions and edges in blue represent negative interactions.

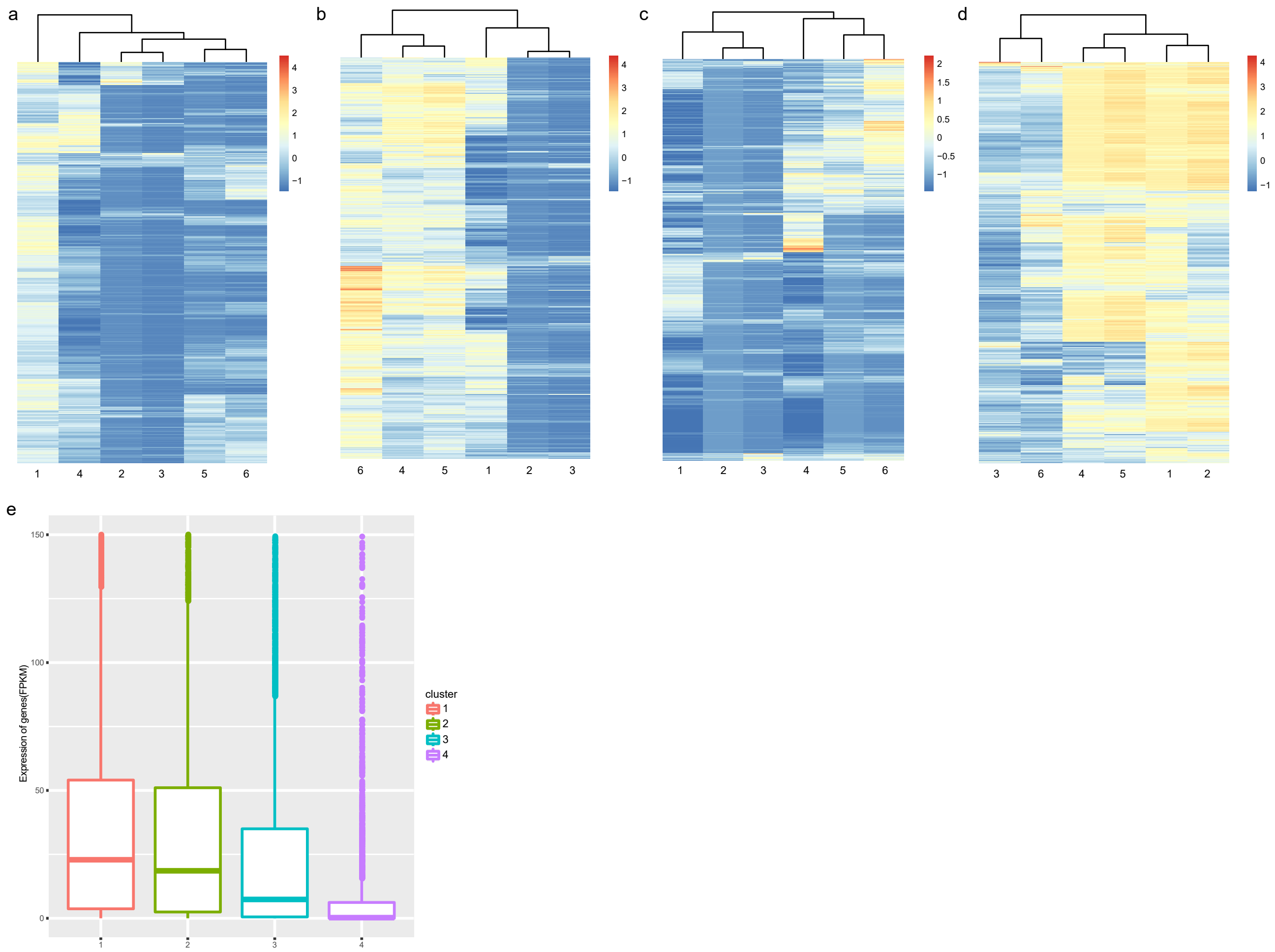

Supplementary Figure S4 The methylation type of the pineapple genome in CK, LE and HE. A-D: the heatmap of cluster 1-4. 1: the CG methylation of gene upstream; 2: the CHG methylation of gene upstream; 3: the CHH methylation of gene upstream; 4: the CG methylation of gene body; 5: the CHG methylation of gene body; 6: the CHH methylation of gene body.

Supplementary Table S1 Summary of iso-seq reads.

Library	reads of insert	five prime reads	three prime reads	poly-A reads	full-length non-chimeric reads	full-length non-chimeric read length(bp)
**<1K**	306,524	224,869(73.36%)	240,144(78.34%)	227,525(74.23%)	184,148(60.08%)	649
**1-2 K**	325,575	284,151(87.28%)	293,958(90.29%)	295,218(90.68%)	257,914(79.22%)	1,835
**2-3 K**	311,218	265,959(85.46%)	277,735(89.24%)	280,311(90.07%)	241,999(77.76%)	2,947
**>3 K**	365,242	305,471(83.64%)	269,573(73.81%)	52,955(14.5%)	41,812(11.45%)	2,810
**Total**	1,308,559	774,999	1,081,410	856,009	725,873	

Supplementary Table S2 Summary of high quality reads and low quality reads.

Library	Cluster type	Total isoforms	Total base(bp)	Mean Quality	Mean isoform length(bp)	Mean Full length coverage	Mean Non Full length coverage
>3 K	High quality	9,967	24,357,387	0.9835	2,444	2.00	2.08
>3 K	Low quality	18,953	55,194,220	0.2314	2,912	1.15	0.96
<1 K	High quality	36,152	21,664,343	0.9468	599	4.02	4.53
<1 k	Low quality	34,417	22,854,299	0.3576	664	1.12	0.84
1-2 K	High quality	40,673	74,763,153	0.9793	1,838	3.40	3.62
1-2 K	Low quality	91,253	166,799,036	0.0672	1,828	1.31	1.21
2-3 K	High quality	35,546	102,935,180	0.9851	2,896	3.40	3.68
2-3 K	Low quality	90,294	271,114,998	0.0976	3,003	1.34	1.23

Supplementary Table S3 Summary of Illumina short reads.

**Sample Name**	**Clean reads**	**Genome map rate**	**Gene map rate**	**Expressed gene**
HE1	30487590	55.15%	40.63%	19197
HE2	30085504	55.09%	41.21%	19073
HE3	29442846	57.28%	41.63%	19156
CK1	26870796	59.65%	43.62%	18497
CK2	29999998	60.18%	43.10%	18775
CK3	27224962	60.87%	43.61%	18732
LE1	29526890	62.19%	44.17%	19101
LE2	29688904	61.42%	43.62%	19300
LE3	26878052	59.63%	43.51%	19067

Supplementary Table S4 The primer for protein coding gene qRT-PCR validation.

Accession Number	Sense Primer	Anti-sense Primer	Amplicon Length (bp)	E(%)	R^2
XM_020227265.1	ATATACGAAGGATACGCTCTC	CCGTCAGGCAACTCATAG	242	100.4	0.999
XM_020252320.1	GACCGCCTTGTTGTGATA	GTGTTCCAGTAGTTCTTGATG	322	100.2	0.999
XM_020252482.1	GCTATATTCGTCGTAGTCAAC	CCTTGTAATCACCTTGGATATG	139	99.7	0.992
XM_020251135.1	CAACAGCAGTCACCTCTAT	CTTCAGCAGCAGAACCTT	146	105.8	0.997
XM_020248230.1	GAGGTTGCCGCTTATGAT	CCACACGCTTATGATTGC	189	106.3	0.993
XM_020233550.1	CTTCTACAGTAACAGGACAATG	GAATATCAGTCACCATCCAGT	191	102.6	0.991
ACMD2_16179	GAGGCGGTTCACTACAAC	GTCGAAGGCTTCCAGTATG	375	97.6	0.999
XM_020256952.1	CATAATCCGACTGCTCTCA	AACTTCTTGCTCCAACTCA	312	102.4	0.995
XM_020256529.1	CCATTGACTACATGAAGGAG	CACACTGCTGAATCTCTAAC	264	108.3	0.999
XM_020233032.1	CCACCACCACAAGATGAG	CGGCTGAGAAGAGATATGAA	290	106.9	0.994
XM_020235999.1	CGACGGAGAAGTGTAAGAG	CGACTATGTGCCTGGTTG	306	102.8	0.999
XM_020254998.1	ACAACCGAGCAGATTCAG	AGATCCGACTCCTCCAAG	250	103.9	0.992
XM_020258797.1	TTCAAGGAGAAGCAGCAG	CGTTGTTGGAATTGGAGAG	220	104.7	0.998
XM_020251774.1	GAATCACCACCGCTGTTG	TCTCCTCATCGGCTTCTC	103	98.6	0.992
XM_020235686.1	TAGCAGGTTCCATCAGTTG	AGCTGTTCGATGATAATCCA	307	99.4	0.995
XM_020242054.1	GTCCTGGATGGCTATGATG	CGCTCGTGTAGATGAAGAT	187	103.5	0.999
XM_020254617.1	GATTCTCAGCTTGCTACCA	GCCACGACTTATTATTATCCTC	487	104.4	0.993
XM_020226779.1	TACAGTAACAGGACAATGGT	GCCAGAAGCAGAGATGAA	237	106.5	0.998

E: PCR PCR efficiencies. R^2: linear correlation coefficient. ACMD2_16179 is the transcript of gene coding protein QAY62772.1.

Supplementary Table S5 The primer for lncRNA qRT-PCR validation.

Accession Number	Sense Primer	Anti-sense Primer	Amplicon Length	E(%)	R^2
**c10180/f1p1/463**	TTTAGGTCGTTCGGGAATA	ACTCAAGTATGGTGTAAGGA	143	98.3	0.999
**c19722/f1p1/1757**	CATCCTCTTCTCACCATACG	CTTCGCACACCAGCATAA	188	99.6	0.999
**c19302/f1p2/2888**	AGCCGCCTTATCACCTTA	CGACTATCCTGAAGAACCAA	498	101.2	0.999
**c44513/f2p2/2734**	GCGTGTAAGTATGGTGGTT	ATGTGGAGTCGTGATGGT	182	99.5	0.992
**c23999/f1p1/1076**	TACTCTTAGGTGGTGAATCG	ATGCTTGTTGACGGTGAA	455	100.3	0.995
**c4177/f1p1/411**	ATTGTAGTCGGCGATGATT	AATTCTCCAGTCACTCTCAA	141	104.6	0.993
**c115298/f2p2/3179**	CCGACTAATGCCGATTCC	CCAGACAAGAAGACGAAGG	190	102.9	0.997
**c17838/f1p1/318**	GAGAATGCCAATGAAGAGG	GCCCACAATTTCATATCACA	144	104.8	0.994
**c123743/f1p1/310**	AGTCACGACCACTACAACA	GCTCCTGCAAACACGAAA	100	106.2	0.997
**c89716/f13p14/516**	GAGTTACGTGCGTACATAC	AGCAATAACAGAGTGAACAG	317	100.5	0.999
**c135159/f1p1/3244**	CAACTGATGGAACCTATGATG	GGTGGCAATAGCATTATGTT	261	101.7	0.995
**c19106/f1p1/2692**	TAACTGCTCAACAACTCTCA	TTCCTCTTCCTCCTCTTCC	463	97.8	0.998
**c21630/f3p2/438**	GGATCGAGTAGCTCTATTAGG	CCGTCCTCTTCAATCAGTT	131	99.3	0.997
**c12262/f3p4/1749**	GCCACGACTTATTATTATCCTC	CCACACTTGCTTCAATCAC	173	106.8	0.996

E: PCR PCR efficiencies. R2: linear correlation coefficient.

Supplementary Dataset S1 Annotation of novel coding transcripts.

Supplementary Dataset S2 Gene information on flowering-related subnetworks.

Supplementary Dataset S3 The DMRs overlapped with genes of flowering related subnetworks.
